# CXCL12-Mediated Guidance of Migrating Embryonic Stem Cell-Derived Neural Progenitors Transplanted into the Hippocampus

**DOI:** 10.1371/journal.pone.0015856

**Published:** 2010-12-31

**Authors:** Nathaniel W. Hartman, Joseph E. Carpentino, Kristi LaMonica, Danielle E. Mor, Janice R. Naegele, Laura Grabel

**Affiliations:** 1 Department of Biology, Wesleyan University, Middletown, Connecticut, United States of America; 2 Program in Neuroscience and Behavior, Wesleyan University, Middletown, Connecticut, United States of America; Universidade Federal do Rio de Janeiro, Brazil

## Abstract

Stem cell therapies for neurodegenerative disorders require accurate delivery of the transplanted cells to the sites of damage. Numerous studies have established that fluid injections to the hippocampus can induce lesions in the dentate gyrus (DG) that lead to cell death within the upper blade. Using a mouse model of temporal lobe epilepsy, we previously observed that embryonic stem cell-derived neural progenitors (ESNPs) survive and differentiate within the granule cell layer after stereotaxic delivery to the DG, replacing the endogenous cells of the upper blade. To investigate the mechanisms for ESNP migration and repair in the DG, we examined the role of the chemokine CXCL12 in mice subjected to kainic acid-induced seizures. We now show that ESNPs transplanted into the DG show extensive migration through the upper blade, along the septotemporal axis of the hippocampus. Seizures upregulate CXCL12 and infusion of the CXCR4 antagonist AMD3100 by osmotic minipump attenuated ESNP migration. We also demonstrate that seizures promote the differentiation of transplanted ESNPs toward neuronal rather than astrocyte fates. These findings suggest that ESNPs transplanted into the adult rodent hippocampus migrate in response to cytokine-mediated signals.

## Introduction

Stem cell-based treatments for neurodegenerative diseases and central nervous system (CNS) injuries are currently in the pipeline. Embryonic stem cell (ESC)-derived neural progenitors (ESNPs) are among the most promising candidate neural cell types under investigation for CNS repair because they retain the potential to proliferate and differentiate into multiple neuronal and glial subtypes following transplantation [1], with the specific outcome dependent upon local environmental cues [2], [3]. As these cells differentiate, they form functional neurons capable of incorporating into the host brain [4].

For effective CNS repair, ESNPs must be directed to sites of damage [5], [6], but little is known about how these cells migrate after transplantation. Effective therapies for widespread white matter damage in diseases like multiple sclerosis may require long-range dispersal of glial progenitors [7], [8]. In contrast, conditions such as spinal cord injury, Alzheimer's disease, Parkinson's disease, stroke, or temporal lobe epilepsy (TLE), may need focal delivery of replacement cells to denervated sites [9]. Therefore, a better understanding of the molecular mechanisms involved in migration and differentiation of ESNPs and their derivatives is essential for successful stem cell-based CNS therapy design.

A number of studies have shown that neural stem cells (NSCs) derived from either the adult CNS or ESCs incorporate into the upper blade of the dentate gyrus (DG) granule cell layer (GCL) and differentiate into dentate granule neurons (DGNs) after transplantation into the adult hippocampus [10]. Previous analysis suggests that transplanted cells disperse passively throughout the site of a neurodegenerative lesion caused by fluid injections into the upper blade of the DG [11], [12]. Whether transplanted NSCs actively migrate in this region has not been well studied. We therefore examined the distribution of transplanted ESNPs after they were deposited in the adult hippocampus in mice that had been subjected to kainic acid (KA)-induced status epilepticus (SE). Seizures may influence migration and/or differentiation through upregulation of stromal derived factor-1α (CXCL12 or SDF-1α), a potent chemokine produced by the meninges and DGNs both during embryogenesis and in the adult hippocampus [13], [14].

CXCL12 signaling via its primary receptor, CXCR4, guides migrating granule neural precursors from the hilus into the DG during development [15], [16]. CXCL12 also acts as a chemoattractant for tangentially migrating GABAergic interneurons within the developing cerebral cortex and hippocampus [17]. In addition, new evidence suggests that CXCL12 is critical for the migration of NSCs from the subventricular zone (SVZ) into the rostral migratory stream (RMS) [18], as well as the migration and proliferation of NSCs engrafted into the spinal cord in a rodent model of multiple sclerosis [19]. Moreover, CXCL12 regulates the migration of both endogenous and transplanted NSCs in stroke models in adult rodents [20], [21]. This chemokine pathway also influences the differentiation of newborn DGNs in the adult hippocampus [22], [23].

We examined the extent and direction of migration of ESNPs transplanted to the adult DG and observed significant movement from the injection sites posteriorly along the upper blade of the DG into sites where the endogenous DGNs degenerate. Expression of CXCR4 by ESNPs suggests that CXCL12 is involved in this process. This hypothesis was supported by our finding that seizures upregulated CXCL12 expression in the hippocampus DG. Moreover, ESNPs showed a chemotactic response to CXCL12, both *in vitro* and *in vivo*. These data suggest that a localized source of CXCL12 may direct the migration of ESNPs towards specific regions of neurodegeneration in the adult hippocampus.

A second goal was to examine whether SE prior to transplantation influenced migration or differentiation of transplanted ESNPs. Seizures have a profound influence on gene expression, synaptic connectivity, and neuronal survival in the hippocampus [24], [25]. Patients with severe chronic TLE show distinctive neuropathological changes including hippocampal sclerosis and cell death throughout Ammon's horn and the dentate hilar region [26], [27]. Similar patterns of neuronal loss are apparent following SE in experimental models [28], [29]. Seizures also induce abnormal upregulation of neurogenesis in the subgranular zone (SGZ) of the human and rodent DG [30], [31], [32], [33] and alter the fates of endogenous NSCs [34]. Our results demonstrate that seizures induce a marked change in the differentiation of ESNPs into neurons rather than astrocytes.

## Materials and Methods

### Cell Culture

YC5 mouse ESC cultures were maintained on a feeder layer of STO fibroblasts, with DMEM (Sigma, St. Louis, MO) supplemented with leukemia inhibitory factor (LIF), 1-thioglycerol and Knockout Serum Replacement (Gibco, Grand Island, NY). Neural progenitors were derived using a modification of a previous protocol [35]. ESCs were differentiated into embryoid bodies in suspension culture in the presence of fetal bovine serum (Atlanta Biologicals, Lawrenceville, GA) and the absence of LIF, then grown in adherent culture using serum-free medium supplemented with insulin, transferrin, selenium (ITS, Gibco), and fibronectin (Sigma). ESNPs were expanded in neural expansion media (DMEM/F12 [Sigma], putrescine [Sigma] and laminin [Sigma]), containing fibroblast growth factor 2 (FGF2, Sigma) and epidermal growth factor (EGF, Sigma) for 5–9 days. For *in vitro* assays and ESNP characterization, cultures of ESNPs were grown in Permanox chamber slides (Thermo Scientific, Rochester, NY). Cells were fixed in 3.7% formaldehyde in 0.1 M phosphate buffer and stained by immunocytochemistry for cell type-specific markers and nuclear staining with Hoechst 33342 (Invitrogen, La Jolla, CA). To test the effects of CXCL12 (Peprotech, Rocky Hill, NJ) or AMD3100 (Sigma), ESNPs grown on Permanox were treated with 200 ng/mL CXCL12 and/or 5 µg/mL AMD3100 for three days with media changes each day.

### Surgical Procedures

This study was carried out in strict accordance with the recommendations in the Guide for the Care and Use of Laboratory Animals of the National Institutes of Health. The protocol was approved by the Wesleyan University Animal Care and Use Committee (Protocol Number: 090093). Eight- to ten-week-old male B6.CB17-Prkdc scid/SzJ mice (Jackson Laboratories) were injected subcutaneously with KA (Sigma, 22.5 mg/kg in saline) to induce SE. Control mice received injections of PBS. Seizures were graded as described elsewhere and attenuated by sodium pentobarbital (50 mg/kg, i.p., Abbott, North Chicago, IL), 10 min after reaching stage 5 seizures, as described previously [28], [36]. Cell death in the hippocampus from single systemic injections of KA in this mouse line has been previously described [2].

ESNPs were transplanted into the DG by means of stereotaxic surgery, 7 days following the induction of seizures by the chemoconvulsant KA. Surgical levels of anesthesia were induced by inhalation of isoflurane (Sigma). The stereotaxic surgery was performed in a rodent stereotaxic frame fitted with an isoflurane gas mask adaptor for mice (Kopf, Tujunga, CA). Coordinates for stereotaxic injections into the hippocampus were based on distance from Bregma; −2.0 mm posterior, −1.95 mm lateral, and −1.9 mm ventral to the pial surface. Approximately 50,000 cells in 1 µl of neural expansion media were injected over a period of 10 min. The needle was then removed and the incision sealed with veterinary-grade tissue adhesive (Vetbond, 3 M). To test migration, osmotic minipumps (Alzet Model 1004, Cupertino, CA) were placed subcutaneously in the intrascapular area one day prior to, or 3 hours following, transplantation. Osmotic minipumps were filled with either 0.1 M phosphate buffer or 50 µg/µL AMD3100 diluted in 0.1 M phosphate buffer. The incision was sealed with veterinary-grade tissue adhesive and wound clips.

### Histological Analyses

Mice were euthanized with a lethal dose of sodium pentobarbital (Nembutal, Abbott Labs, North Chicago, IL) followed by transcardial perfusion with a heparinized phosphate (PO_4_) buffer solution containing 10% sucrose, then 4% PFA in 0.1 M PO_4_. Brains were cryogenically protected using graded sucrose solutions to 30% in 0.1 M PO_4_. Brains were then embedded in tissue freezing medium (Triangle Biomedical Sciences, Durham, NC) and frozen at −80°C. Cryostat sections were cut at 12-µm intervals along the coronal plane and mounted onto Superfrost Plus slides, briefly dried on a slide warmer, and then stored at −80°C until immunohistochemical analyses were performed.

### Immunostaining

ESNPs were cultured in chamber slides, fixed in 3.7% formaldehyde for 10 minutes at room temperature, rinsed in PBS, and permeabilized in 0.1% Triton X-100/PBS for 10 minutes at room temperature. After a blocking step (2% bovine serum albumin in 0.1% Triton X-100/PBS) for 1 hour at room temperature, primary antibodies were placed in each chamber overnight at 4°C. For all immunostaining experiments, a negative control experiment was performed in which the primary antibody was omitted. For immunohistochemistry, brain sections were briefly rehydrated in PBS. After blocking (2% bovine serum albumin, 10% normal goat serum in 0.1% Triton X-100/PBS) for 1 hour at room temperature, primary antibodies were applied in blocking buffer and incubated overnight at 4°C. Antibodies were diluted as follows: mouse anti-nestin, 1∶1,000 (Chemicon, Temecula, CA); mouse anti-TuJ-1, 1∶1,000 (Covance); rabbit anti-doublecortin, 1∶1,000 (Sigma); mouse anti-Prox1, 1∶200 (Chemicon); rabbit anti-glutamine synthetase 1∶1,000 (Sigma); mouse anti-NeuN 1∶500 (Chemicon); rabbit anti-CXCR4 1∶200 (Torrey Pines, East Orange, NJ); rabbit anti-CXCL12 (Abcam, Cambridge, MA). After rinsing, secondary antibodies were then applied, diluted 1∶1,000 (Alexa-Fluor 568- or 647-conjugated goat anti-mouse IgG, Alexa-Fluor 568- or 647-conjugated goat anti-rabbit IgG, Alexa-Fluor 568-conjugated goat anti-rat IgG; Invitrogen, La Jolla, CA) in blocking buffer for 1 hour at room temperature. Enhanced yellow fluorescent protein (EYFP)-positive cells were visualized by using either rabbit anti-green fluorescent protein (GFP) diluted 1∶1,000 or mouse anti-GFP diluted 1∶1,000 (Invitrogen), followed by Alexa-Fluor 488. Cell nuclei were then labeled with Hoechst 33342 (Molecular Probes, Eugene, OR), diluted 1∶10,000, and sections were mounted in gelvatol.

### *In Vitro* Migration Assay

The top surfaces of 8 µm-pore transwells (Corning, Corning, NY) were coated with 2 µg/mL laminin and ESNPs were seeded in DMEM/F12 medium at a density of 150,000 cells per transwell. Transwells were inserted into 24-well culture plates on top of 0.5 mL of DMEM/F12 containing varying concentrations of CXCL12 recombinant peptide (Peprotech, Rocky Hill, NJ). In order to block CXCR4 receptors, ESNPs were first incubated in varying amounts of AMD3100 (Sigma) for 30 min. at room temperature, washed in PBS and then resuspended in DMEM/F12. The transwells were allowed to incubate for 18 hours at 37°C, washed three times in PBS, fixed in 3.7% formaldehyde and stained for nuclei with Hoechst. Transwell membranes were removed and the underside of the membrane was imaged and nuclei counted using NIS-Elements (Nikon, Melville, NY) software.

### Quantitative and Semi-Quantitative PCR

Reverse transcription (RT) and PCR reactions were performed as previously described [37]. Briefly, total RNA was extracted from ESCs, ESNPs, E14.5 whole brain and whole adult hippocampal lysates using Ultraspec-RNA (Biotecx Laboratories, Inc., Houston, TX). RNA was treated with RNAse free DNase I (Invitrogen) and reverse-transcribed with MMLV-RT (Invitrogen). cDNAs were amplified with various primers using Taq-ReadyMix (Sigma). β-actin was used as a loading control. Primer sequences for semi-quantitative PCR were as follows, β-actin: 5′- GGCCCAGCGCAAGCGAGGTATCC -3′ and 5′- ACGCACGATTTCCCTCTCAGC -3′, CXCL12: 5′- GCTAAGGTTTGCCAGCATAAAGAC -3′ and 5′- ACAGCGGTGAGAAGCGGAAGTCAG -3′, CXCR4: 5′- ACAGGTACATCTGTGACCGCCTTT -3′ and 5′- TGCTCTCGAAGTCACATCCTTGCT -3′, and CXCR7: 5′- CTGAGGTCACTTGGTC -3′ and 5′- GATGGAGAGATAGCGGTCCAC -3′. Cycle number for each primer pair was determined individually so that amplification was in the exponential range and had not reached a plateau.

Real-time RT-PCR was performed using 100 ng of total cDNA per reaction. cDNA was combined with primer/probe sets for CXCL12 and GAPDH and TaqMan Gold RT-PCR Master Mix and run on an ABI 7300 (all Applied Biosystems, Foster City, CA). The RT-PCR profile consisted of one cycle at 50°C for 2 min and 95°C for 10 min, followed by 40 cycles at 95°C for 15 s and 60°C for 1 min. Results of the real-time RT-PCR were normalized to GAPDH. To assess the difference in mRNA levels, the data were analyzed using the 2-CT method.

### Quantification of Graft Size and Migration

Transplant grafts were measured by sampling brain sections every 120 µm through the hippocampus and the injection site. Area measurements of the grafts were calculated using NIS-Elements software (Nikon Instruments Inc., Melville, NY). Volumetric calculations of the grafts were calculated from the cross-sectional areas of the grafts multiplied by the distance between sampled sections. Statistical analysis was performed using Student's T-test and ANOVA using Microsoft Excel. Significance was determined as having a *p* value less than 0.05.

## Results

### ESNP Differentiation *In Vitro*

YC5 mESCs were differentiated into ESNPs using a modification of a standard embryoid body-based protocol, using defined media [35]. At the time of transplantation, over 90% of the cells expressed the NSC marker nestin, and less than 10% expressed the immature neuronal marker β-III tubulin (Fig. S1A–C). The transplant population also expressed additional markers for neural precursors (Sox1) as well as anterior (Otx1) and ventral forebrain markers (Mash1, Nkx2.1) and dorsal markers (Pax7), demonstrating the presence of broad regional identities in these cultures (Figure S1D).

### Injection Site Location Influences ESNP Dispersion in the Adult Hippocampus

**Figure 1:**
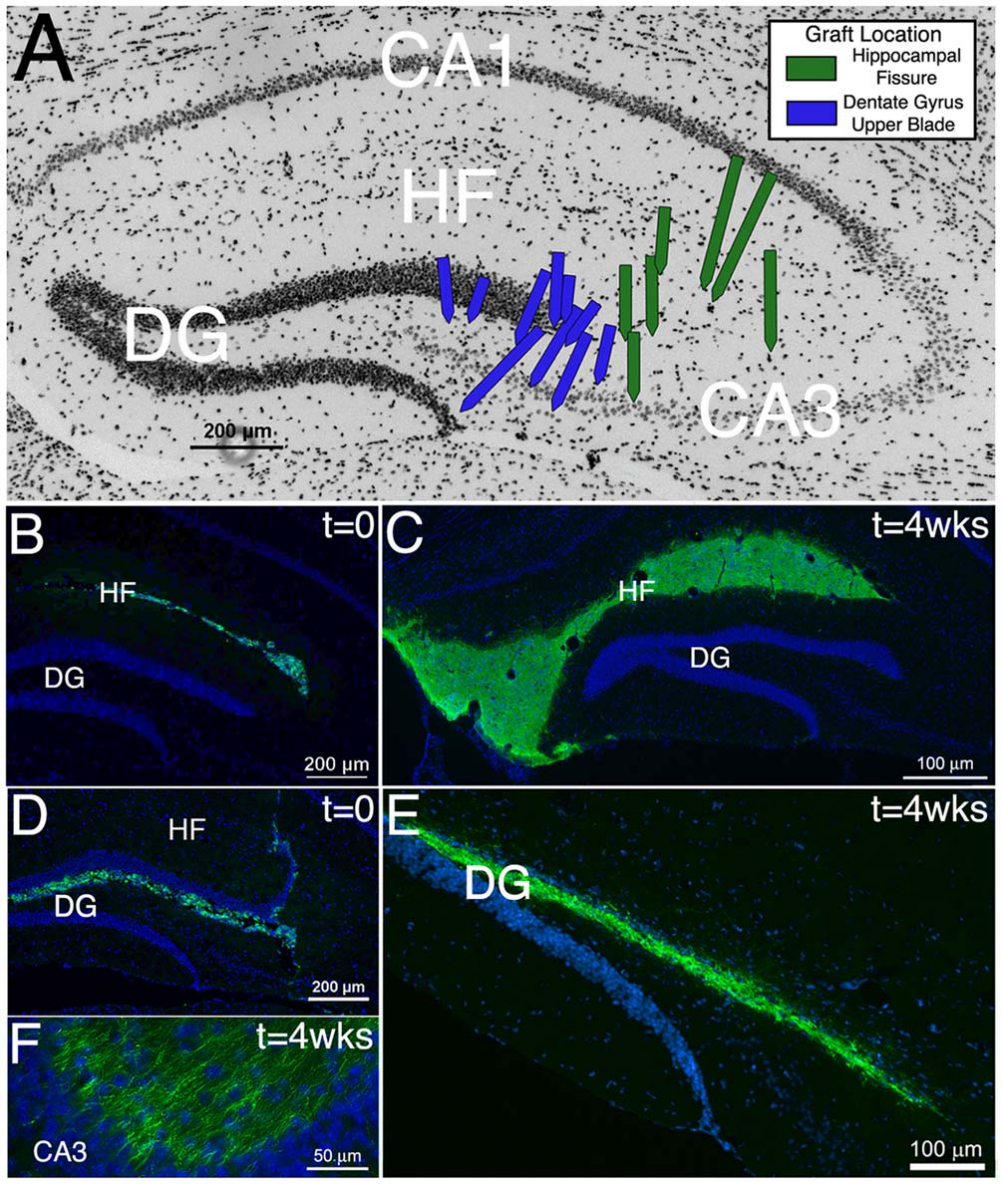
Injection site affects ESNP dispersal in the hippocampus. (A) Schematic of 17 representative transplants shows both the needle track (arrows) and placement of ESNPs 4 weeks following surgery in either the hippocampal fissure (HF, green arrows) or the upper blade of the DG (DG, blue arrows). (B) ESNPs (green) injected into the hippocampus disperse through the HF and persist at 4 weeks following transplantation (C). (D) When the injection site is closer to the DG, ESNPs are dispersed along the upper blade, where they persist at 4 weeks (E) and can send processes contacting cells in the CA3 pyramidal layer (F). Cell nuclei are counterstained blue with Hoechst.

Previously, we reported that ESNPs survive and differentiate into neurons in the DG of mice, following KA-induced seizures [2]. To examine whether dispersion of ESNPs is affected by the site of injection, we compared graft location following injections into different locations. When the stereotaxic injection sites were located closer to CA3 (green arrows, Figure 1A), ESNPs tended to settle in the hippocampal fissure (HF) immediately following injection (Figure 1B), and remain there up to 4 weeks following transplantation (Figure 1C). ESNPs transplanted to the HF survived but did not migrate into the hippocampus from the fissure (Figure 1C). When the needle track was close to, or pierced the DG (blue arrows, Figure 1A), the ESNPs were mainly localized within the infragranular zone beneath the upper blade of the DG shortly after transplantation (Figure 1D). After 4 weeks however, the transplanted cells had dispersed through the upper blade of the DG (Figure 1E). To determine whether the dispersion was linked to neurodegeneration within the granule cell layer of the DG, we examined NeuN+ neurons in the GCL at different times after injections were made. Over the course of a week following ESNP transplantation, we observed a prominent loss of NeuN+ cells in the GCL, particularly in the upper blade (Figure S2). The ESNPs infiltrated the upper blade by 3 days after transplantation and by 4 weeks had filled in the sites in the GCL where endogenous DGNs underwent degeneration (Figure S2). These results show that transplanted ESNPs incorporate into lesions in the DG of the hippocampus, in agreement with previous studies using fetal NSCs [10].

### Migration of ESNPs in the DG

**Figure 2:**
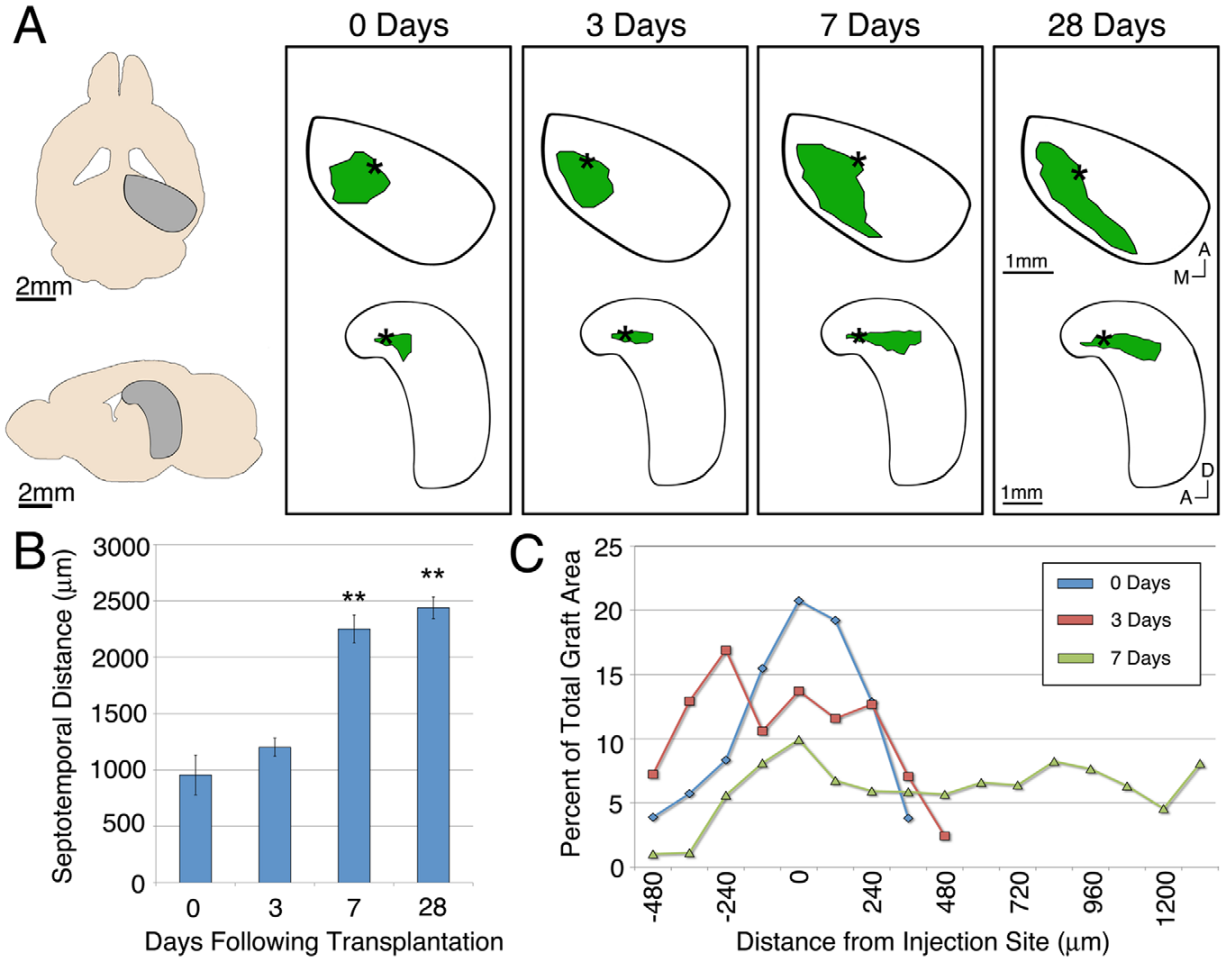
ESNPs disperse along the anterior-posterior axis of the DG upper blade following transplantation. (A) Horizontal and lateral reconstructions of representative transplants to the upper blade show the initial dispersion of ESNPs (green) from the injection site (*). The graft then extends along the upper blade in the posterior direction over the course of a week. (B) Quantification of the graft extent along the septotemporal axis shows that the maximum distance is reached by one week following transplantation. (C) Analysis of graft distribution shows that transplanted cells initially cluster close to the injection site and then are more evenly distributed as the graft extends to the posterior hippocampus over the course of 7 days. * and ** denote p values less than 0.05 and 0.01 by Student's t-test, respectively.

We next asked whether transplanted ESNPs showed directional migration within the DG. To address this question, we reconstructed graft locations in the DG at different time points. When cells were transplanted into the upper blade in the dorsal hippocampus (asterisks, Figure 2A), ESNPs dispersed posteriorly over the course of one week, along the septotemporal axis of the hippocampus (Figure 2A). Cells in these grafts rarely migrated away from the upper blade. Conversely, ESNPs transplanted into other regions of the hippocampus were never observed to disperse into the infragranular zone (data not shown). The ESNP grafts in the upper blade reached a maximum septotemporal extent by 7 days following transplantation, with a small significant increase at 28 days (Figure 2B). During this period, ESNPs became more evenly distributed (Figure 2C). These observations, demonstrating that ESNPs transplanted into the SGZ or hilus dispersed within the infragranular zone, suggest that an active process mediated by local signals within the DG may regulate ESNP migration.

### ESNPs Migrate in Response to a Source of CXCL12

**Figure 3:**
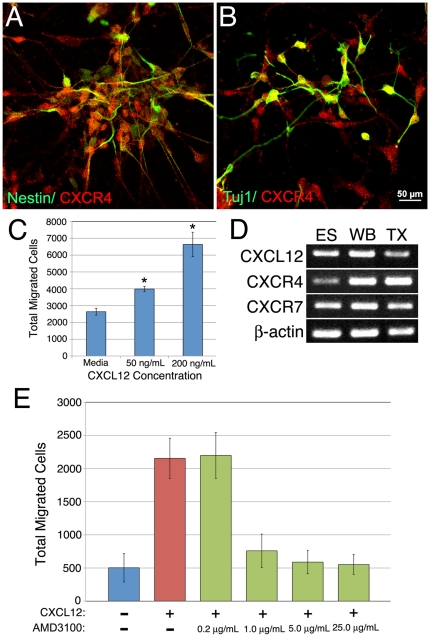
CXCL12 induces migration through the CXCR4 pathway in ESNPs. ESNPs expressing nestin (green, A) and the immature neural marker β-III tubulin (B, green) co-labeled with the CXCL12 receptor, CXCR4 (A and B, red). (C) In an *in vitro* trans-well migration assay, CXCL12 induced migration across the membrane in a dose-dependent manner (p<0.01 ANOVA). (D) Expression of CXCL12 mRNA as well as mRNA for both CXCL12 receptors, CXCR4 and CXCR7, was present in ESCs (ES), E12.5 whole brain lysates (WB) and transplanted ESNPs (TX). (E) In an *in vitro* trans-well migration assay, blockade of the CXCR4 receptor with a small pharmacological inhibitor, AMD3100, was sufficient to eliminate CXCL12-induced migration (p<0.01, ANOVA). * denotes p values <0.05 by Student's t-test.

To determine whether ESNP dispersion is an active process guided by established chemoattractants, we investigated the role of CXCL12, a known chemokine expressed in the adult brain [38]. In culture, ESNPs, as well as ESC-derived immature neurons, expressed CXCR4, a receptor for CXCL12 required for CXCL12-mediated chemotaxis (Figure 3A, B). Moreover, transwell migration assays demonstrated clearly that ESNPs migrated in a dose-dependent manner towards a source of CXCL12 (Figure 3C). These results suggest an active migration process that is dependent upon CXCL12.

To further investigate CXCL12-mediated guidance mechanisms, we next examined expression of CXCR4 and CXCR7, the two receptors that bind CXCL12 [39]. YC5 ESCs and ESNPs express mRNA for both the chemokine and its receptors (Figure 3D). To determine whether CXCL12-induced migration is mediated via CXCR4 receptors, we blocked CXCR4 receptors with a small pharmacological inhibitor, AMD3100 [40]. This inhibitor effectively reduced ESNP migration towards an *in vitro* source of CXCL12 (Figure 3E). These data suggest that CXCL12-mediated migration of ESNPs requires the function of CXCR4. This result, however, could be due to altered proliferation or differentiation of ESNPs, so we next determined whether CXCL12 affects these properties. *In vitro* treatment with CXCL12 did not increase phospho-histone H3 levels or alter nestin or β-III tubulin staining (Figure S3). We therefore conclude that CXCL12 is a candidate molecule for regulating ESNP migration in the adult hippocampus.

### CXCL12 is Upregulated Following KA-Induced Seizures

**Figure 4:**
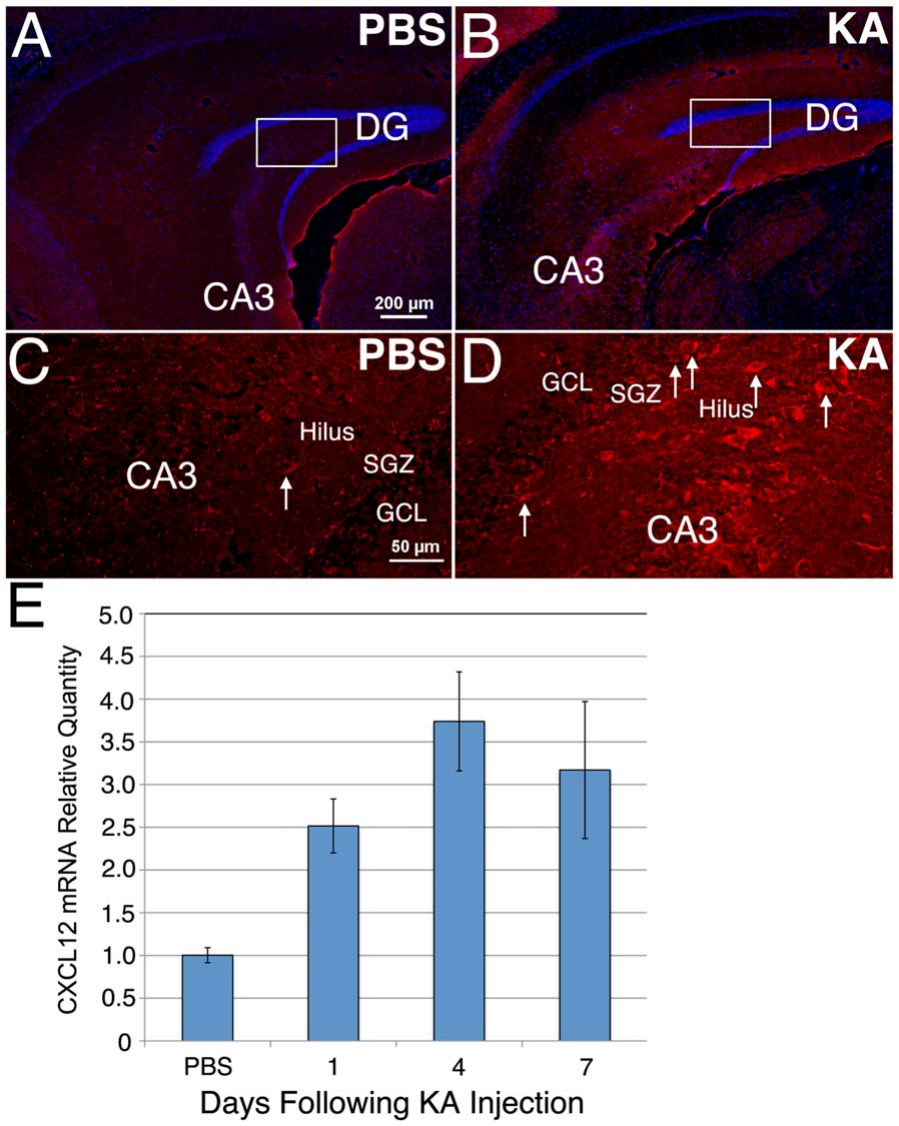
CXCL12 expression increases in the DG following KA-induced seizures. Immunofluorescent staining revealed a low level of CXCL12 expression in the hippocampus prior to seizures (red, A). Expression was markedly increased 7 days following KA-induced seizures (red, B). Arrows denote examples of CXCL12-immunoreactive cells. (C, D) Confocal imaging of the boxed areas in A and B showed a dramatic increase of CXCL12 staining of cells in the SGZ as well as the hilus of the DG and the CA3 pyramidal layer. (E) Quantitative RT-PCR analysis of whole hippocampal lysates showed a 2.5 to 3.5-fold increase in CXCL12 message (three mice per time point, p<0.01 ANOVA).

We next asked whether seizures impacted the expression of CXCL12 in the hippocampus. At the time of injection of ESNPs into the DG, post-seizure expression of CXCL12 was elevated in the DG and CA3, compared to control mice that did not experience prior seizures, based upon immunohistochemical analysis (Figure 4A, B). The most pronounced increase in CXCL12 expression over basal levels was observed in the SGZ and hilus of the DG (arrows, Figure 4D). To further investigate the role of seizures in induction of CXCL12, levels of mRNA were measured after KA-induced seizures. CXCL12 mRNA levels from whole hippocampal lysates increased approximately 3-fold within one day following KA injection and remained elevated 7 days later (Figure 4E). These data demonstrate that seizures augment CXCL12 expression in the DG.

### AMD3100 Inhibits the Migration of Transplanted ESNPs

Based on our *in vitro* migration data and the expression of CXCL12 in the DG, we hypothesized that CXCL12 guides the migration of ESNPs through the degenerating upper blade of the DG following transplantation. To test this hypothesis, we blocked CXCR4 activity by infusing mice with AMD3100 via osmotic minipumps implanted subcutaneously. Previous studies showed that AMD3100 crosses the blood-brain barrier and exerts physiological effects in the CNS [41].

**Figure 5:**
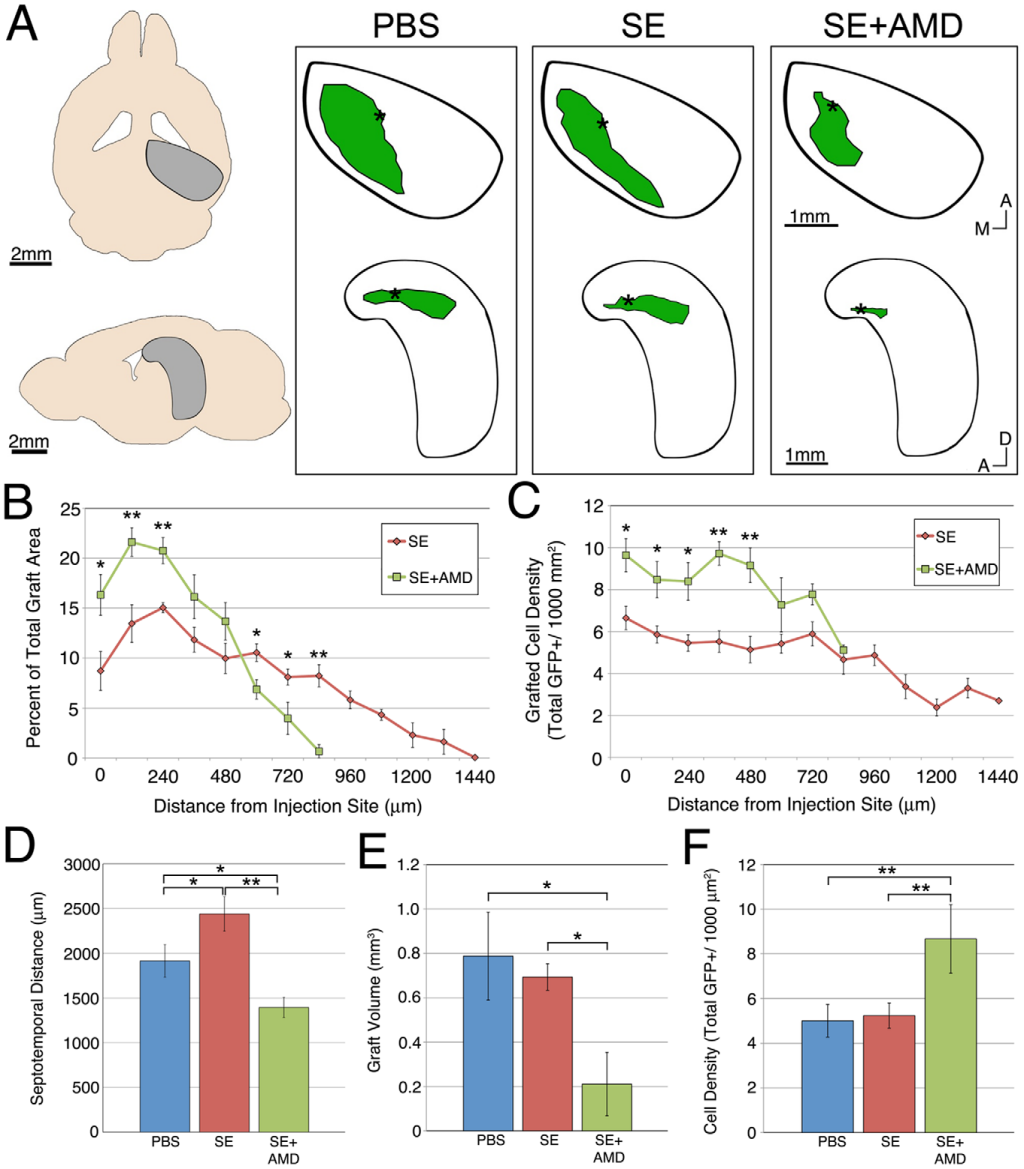
AMD3100 blocks the migration of ESNPs along the upper blade of the DG. (A) Horizontal and lateral reconstructions of representative grafts in the upper blade 4 weeks following transplantation to the DG revealed a truncated posterior extent of the grafts in mice with prior seizure experience and minipumps infusion of AMD3100 (SE+AMD), as compared to mice that only received KA injections (SE). Additionally, mice that did not experience seizures prior to transplantation (PBS) showed comparable graft migration in the posterior direction, compared to the SE group. (B) The graft distribution in the SE+AMD group was less dispersed, and cells remained closer to the injection site compared with the transplants in the SE group. (C) The density of grafted cells in the SE+AMD group was significantly greater near the injection site, compared with cell densities in the SE group. Both the septotemporal extent (D) and average volume (E) of the grafts in both the PBS and SE groups were significantly greater than that of the SE+AMD group. (F) Average cell density of the SE+AMD group was much greater in the SE+AMD groups than either the PBS or SE groups. * and ** denote p values less than 0.05 and 0.01 by Student's t-test, respectively.

ESNPs were transplanted either into mice with no prior seizure experience (PBS) or mice that had received systemic KA injection (SE), and a subset of these mice also received infusions of AMD3100 (SE+AMD). In both the PBS and SE groups not treated with AMD3100, reconstructions of cells grafted to the DG showed a large posterior extension along the upper blade in the septotemporal axis. There was a slight increase in this extension in the SE group versus the PBS group. These data suggest that the upregulation of CXCL12 by seizures (Figure 4) in the adult hippocampus can increase migration of transplanted ESNPs along the septotemporal axis. In SE mice treated with AMD3100, the extent of the grafts was truncated along this axis (Figure 5A). In addition, a significant portion of the grafts in the SE+AMD group clustered near the injection site when compared with controls (58.7% vs. 37.2%, Figure 5B). The AMD3100-induced reduction in migration is consistent with a role for CXCL12 *in vivo* (Figure 5D).

An alternative explanation of the data could be that AMD3100 infusion led to truncation of the total graft volume due to toxic effects on the transplanted cells (Figure 5E). To evaluate this, we next assessed whether treatment with AMD3100 affected ESNP survival in culture. *In vitro* treatment with AMD3100 did not increase apoptosis nor did it alter the extent of differentiation (Figure S4). In addition, the average cell density of the transplants in the SE+AMD group was significantly higher than controls (p<0.01, Figure 5F), suggesting the cells remain clustered around the injection site with AMD treatment, unable to migrate. Consistent with this hypothesis, the largest difference in density between the SE and AMD3100-treated groups was in the sections closest to the injection site (Figure 5C). The observations that AMD3100 does not adversely affect ESNP survival *in vitro*, and that ESNPs are more densely clustered near the injection site, are consistent with the interpretation that AMD3100 infusion inhibits ESNP migration in the DG, implicating a role for CXCL12 as a chemoattractant for ESNP migration.

### ESNPs Differentiate in the Upper Blade of the DG

**Figure 6:**
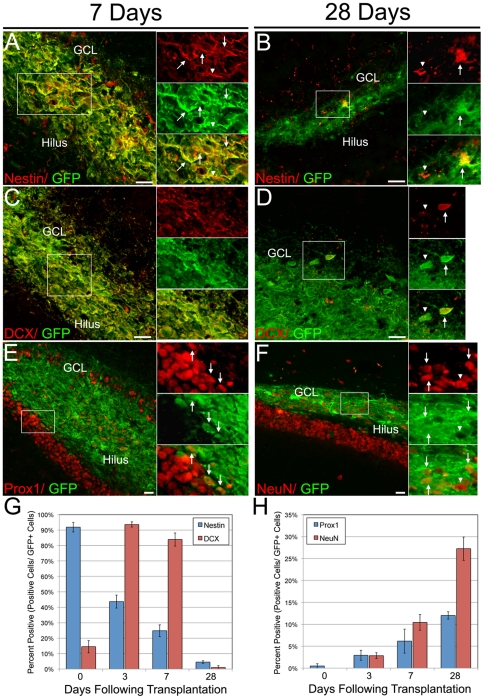
ESNPs differentiate in the upper blade of the DG. ESNPs were transplanted into the upper blade of the DG and mice were sacrificed immediately following transplantation, or 3, 7 (A, C, E) and 28 (B, D, F) days following transplantation. The NSC marker nestin (red, A, B) and the migratory neuroblast marker doublecortin (DCX, red, C, D) showed reduced expression by 4 weeks following transplantation. (G) Quantification of Nestin and DCX expression in transplanted cells revealed a decrease of the NSC marker Nestin following transplantation and a peak of DCX expression at 3 days following transplantation. The dentate granule-specific marker Prox1 (red, E) and the pan-neural marker NeuN (red, F) showed little to no expression initially (0 days), but expression increased up to 28 days following transplantation (H).

Seizure activity leads to dynamic changes in the hippocampus, resulting in a transient increase in neurogenesis in the DG [32]. These observations suggest that seizures might affect ESNP cell fates after transplantation. We first examined the temporal pattern of differentiation of ESNPs transplanted to the DG in animals that had experienced prior seizures. Within one week following transplantation into the DG, a majority of the transplanted ESNPs showed loss of nestin expression, suggesting that they had differentiated (Figure 6A, G). By 4 weeks after transplantation, little nestin expression was observed in the grafted cells (Figure 6B). Concurrently, transplanted cells showed increased expression of doublecortin (DCX), a marker for migrating neuroblasts, peaking 3 to 7 days following transplantation (Figure 6C, G). As observed for nestin, DCX expression was downregulated with longer survival periods of 4 weeks (Figure 6D), suggesting that ESNPs differentiate in the adult DG and upregulate DCX, consistent with a migratory phenotype (Figure 6G). Further differentiation of ESNPs towards DGNs coincided with expression of Prox1 (Figure 6E), a marker of endogenous neural precursors of the SGZ and of mature DGNs in the GCL of the DG [42]. Prox1 expression in the ESNP-derived cell population increased between 1 and 4 weeks (Figure 6H). Concomitant with Prox1 expression, the mature pan-neural marker NeuN begins to be expressed at 1 week after transplantation and levels increased up to 4 weeks following transplantation (Figure 6F, H). In addition, a portion of the grafted cells sent projections toward the pyramidal cells of CA3, (Figure 1E, F). Taken together, these data suggest that in mice subjected to SE, a high proportion of ESNPs transplanted to the DG differentiate rapidly into migratory neuroblasts, and subsequently give rise to DGNs.

### Seizure Experience Alters the Differentiation of ESNPs in the DG

**Figure 7:**
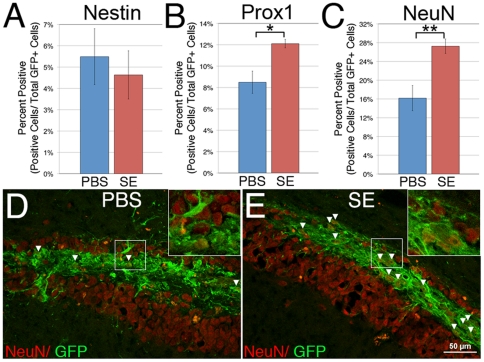
Prior seizure experience increases neuronal differentiation of transplanted ESNPs in the DG. (A) No significant difference is observed in the NSC marker nestin between either the PBS or SE groups. (B) An increase in the dentate specific marker Prox1 is also observed in cells injected into mice with prior seizure experience. (C) Quantification of NeuN expression shows that prior seizure experience leads to a 40% increase in NeuN expressing cells as compared to PBS controls. More grafted cells (green, arrowheads) express the pan-neuronal marker NeuN when transplanted to the DG of mice that had experienced KA-induced seizures (E) than mice that had only received PBS injections (D). * and ** denote p values less than 0.05 and 0.01 by Student's t-test, respectively.

Seizures upregulate a number of factors that increase endogenous neurogenesis in the DG [34], [43]. While our data suggest that elevated levels of CXCL12 following KA-induced seizures do not alter the migration of transplanted cells, increased CXCL12 or other seizure dependent changes may affect the differentiation of transplanted cells. Therefore, we investigated whether prior seizure experience affected the fates of transplanted ESNPs. Expression of the NSC marker nestin in our ESNP grafts was not significantly altered by prior seizures (Figure 7A). However, there was an increase in the expression of the dentate-specific marker Prox1 in the SE group, as compared to the no seizure control group (Figure 7B). In addition, the percent of transplant-derived neurons increased by as much as 40% in mice that had prior seizure experience (Figure 7C). More transplanted cells expressed NeuN throughout the graft area in the SE group than in the PBS group (Figure 7D, E). In addition, grafted cells in the control group expressed a higher level of the astrocyte specific protein glutamine synthetase than either the SE or SE+AMD groups, suggesting that without prior seizures, grafted ESNPs are more likely to differentiate into glial cells than neurons (Figure S5).

## Discussion

The findings of the present study show that ESNPs transplanted into the hippocampus have a stereotypical pattern of dispersion when they are transplanted into the HF or infragranular zone of the DG, in each case likely following paths of least resistance established by injection under pressure. In contrast, we observed that ESNPs generally avoided other subfields of the hippocampus. Within the confines of the DG, transplanted ESNPs migrated within the first week along the upper blade toward the posterior hippocampus, replacing lesioned cells in the GCL over an expanse of 2 mm. Numerous studies have documented the extensive migration of SVZ neural progenitors through the RMS into the olfactory bulb [44], [45]. Recent analysis shows that the chemokine CXCL12 plays a critical role for NSC entry into the RMS [46]. Within the DG, newly born endogenous neurons migrate short distances from the SGZ into the GCL, where they mature into DGNs [47]. Future studies will determine whether endogenous SGZ progenitors can migrate in an anterior-posterior manner along the DG, in the presence or absence of a localized lesion.

Our findings suggest that transplanted ESNPs incorporate into sites where endogenous DGNs undergo degeneration in the upper blade of the dorsal hippocampus. Because granule cells in the septal/dorsal hippocampus receive synaptic information from the entorhinal cortex, and play a crucial role in spatial memory [48], patients with intractable TLE may suffer significant memory losses after ablation of an epileptic focus in the DG. Young DGNs may be especially important for memory consolidation [49]. Migration of ESNPs following transplantation to the upper blade may require the death of the endogenous DGNs, as the migration of transplanted ESNPs through the upper blade correlates with the degeneration of the endogenous DGNs.

Targeting ESNPs to specific zones along the septotemporal axis of the hippocampus could provide an effective therapeutic strategy for hippocampal repair. Our studies suggest that at least part of the mechanism for guiding new cells to their final position in the granule cell layer is the expression of CXCL12, as ESNPs show a directional migration toward a source of this chemokine *in vitro*, and their migration is truncated *in vivo* after infusion with AMD3100, an inhibitor of CXCR4. Our data demonstrate that this chemokine could originate from sources in the hippocampus as well as the transplant population. Previous studies have shown increases in inflammatory cytokines following trauma that may contribute to the migration of transplanted ESNPs [50]. Additionally, CXCL12 upregulation following seizures may promote a slightly more robust migratory event. Alternatively, seizures may exacerbate the damage from fluid injection to the GCL, allowing ESNPs to migrate more extensively.

### Differentiation of ESNPs

We previously reported that subsets of ESNPs grafted to the neurogenic region of the DG expressed Prox1, a granule neuron-specific marker; however, when transplanted to the fimbria, a fiber tract of the hippocampus, ESNPs preferentially differentiated into oligodendrocytes [2]. Others have reported that neural progenitors from adult or fetal tissue can also differentiate into neurons when transplanted into the DG [51], [52]. The SGZ stem cell niche is tightly controlled by growth factors, such as sonic hedgehog, VEGF and BDNF [43], [53], [54], [55]. Under homeostatic conditions, radial NSCs slowly proliferate and give rise to a faster proliferating intermediate astrocyte-like stem cell in the SGZ [56]. As these cells differentiate and migrate into the GCL, they express the migrating neuroblast marker DCX as well as Prox1 [57]. Once these neuroblasts reach their final position in the GCL, they extend a dendritic arbor to the molecular layer and mossy fiber axons to targets in CA3.

Progression from astrocyte-like NSC to DGN during adult neurogenesis in the SGZ takes approximately one month [58]. In the transplanted population, we observed rapid differentiation, with a decline in nestin-expressing ESNPs within 1 week following injection into the DG. In addition, DCX labeled a majority of grafted cells by 3 days following transplantation, and then was rarely detected after 4 weeks. This pattern of expression is consistent with our migration data that demonstrate little posterior movement within three days, but significant migration at one week (Figure 3B). While it takes weeks under normal conditions for newborn cells to mature into neurons in the adult DG [59], approximately 10% of our grafted cells express the pan-neural marker NeuN by 7 days following transplantation. This may be due to the presence of more differentiated cells in the transplant population, of which 16% express DCX. Alternatively, the post seizure environment may induce more rapid differentiation of both endogenous and ESC-derived progenitors.

In addition to accelerated neurogenesis, KA-induced seizures altered the differentiation of ESNPs by promoting neuronal, rather than glial, phenotypes. It is well established that rodent models of TLE initially augment the rate of neurogenesis in the hippocampus [32], [60]. Many factors that promote the proliferation and neuronal differentiation of SGZ stem cells are upregulated following seizures. For example, BDNF injection alone is sufficient to increase hippocampal neurogenesis [61]. In addition, seizures can lead to microglial invasion and reactive gliosis, a potential source for inflammatory cytokines [62]. Moreover, microglia may protect NSCs from factors such as interferon gamma, that suppress proliferation [63]. Recent studies have shown that microglial invasion coincides with the increased expression of cytokines in the DG following pilocarpine administration [64]. Our data corroborate earlier studies demonstrating that CXCL12 expression is increased following status epilepticus, but this increase may be transient without spontaneous recurrent seizures. In addition to seizure-induced changes in the hippocampal environment, the degeneration of the upper blade following fluid injection may increase the expression of factors that may influence the fates of transplanted cells. While previous studies have not documented increases in neurogenesis following dorsal hippocampal lesions in the DG, transient increases in NSC proliferation are observed in similar models of traumatic brain injury [65]. These factors may collectively instruct ESNP differentiation into neurons following transplantation to the DG.

Here, we show that ESNPs transplanted into the DG of the hippocampus migrate along the upper blade. This migration is mediated by the chemokine CXCL12, which is upregulated by seizures. Furthermore, prior seizure experience promotes the differentiation of transplanted cells into neurons. These findings have implications for cell-based therapies in patients with intractable severe seizures. Our results show that ESNPS are recruited to sites of damage. Improper migration of transplanted cells could lead to ineffective treatment or exacerbation of the disease phenotype. Therefore, identifying key regulatory molecules for guiding migration of transplanted cells will lead to more effective stem cell-based therapies for neurodegenerative diseases, traumatic brain injury, and epilepsy.

## Supporting Information

Figure S1 Characterization of ESNPs. Prior to transplantation, ESNPs expressed robust nestin (red, A) and β-III tubulin staining (red, B). (C) Quantification of immunofluorescent staining showed that over 92% of transplanted ESNPs expressed nestin, with fewer than 10% of the cells adopting neural fates, as shown by expression of the immature neuronal marker β-III tubulin. (D) RT-PCR analysis of YC5 embryonic stem cells (ES), E12.5 whole brain lysates (WB) and transplanted ESNPs (TX) showed that transplanted cells expressed markers for early NSCs (Sox1, Otx2), ventral NSCs (Mash1, Nkx2.1) and pluripotency (Oct4), albeit at a lower levels than ES cells. Primer sequences were as follows: Oct4 5′- CTCGAACCACATCCTTCTCT -3′ and 5′- GGCGTTCTCTTTGGAAAGGTGTTC -3′, Sox1: 5′- AATCCCCTCTCAGACGGTG -3′ and 5′- TTGATGCATTTTGGGGGTAT -3′, Otx2: 5′- AGGAGCTGAGTCGCCACCTC -3′ and 5′- GTAGCCCAGGGAGGGATGCA -3′, Emx1: 5′- AGCGACGTTCCCCAGGACGGGCTGC -3′ and 5′- CTGAGGTCACTTGGTC -3′, Pax7: 5′ – CCGTGTTTCTCATGGTTGTG -3′ and 5′- GAGCACTCGGCTAATCGAAC -3′, Mash1: 5′- CGACAGGACGCCCGCCTGAAAG -3′ and 5′- CTCGTCCTCTCCGGAACTGATG -3′, Nkx2.1 5′- AACAGCGGCCATGCAGCAGCAC -3′ and 5′- CCATGTTCTTGCTCACGTCC -3′, and Lhx6: 5′- CGACGACATCCACTACTCTCCGT -3′ and 5′- CAAGCTGAATTCGCCATTGCTCC -3′.
(TIF)

Figure S2 Endogenous DGNs degenerated within one week following ESNP transplantation to the upper blade. (A,B) Immediately following transplantation, NeuN+ neurons (red) in the upper blade were intact, and transplanted ESNPs (green) were clustered at the site of injection. (C,D) Within three days, significant degeneration of endogenous neurons in the upper blade was observed. (E,F) By one week, transplanted ESNPs began to express NeuN (arrows, inset) and degeneration of the upper blade continued. (G,H) By four weeks following transplantation, more engrafted cells were expressing NeuN (arrows, inset), and no more degeneration was observed. Nuclei are counterstained by Hoechst (blue).
(TIF)

Figure S3 CXCL12 did not alter the proliferation or differentiation of YC5 ESNPs. YC5 ESNPs were treated with 200 ng/mL CXCL12 for three days, fixed and stained for markers of proliferation and differentiation. (A) Quantification of phospho-histone H3 revealed no significant difference between CXCL12 treated and untreated ESNPs. (B) Quantification showed similar levels of nestin and β–III tubulin staining after treatment with CXCL12 as compared to controls.
(TIF)

Figure S4 AMD3100 did not alter the differentiation or survival of ESNPs. YC5 ESNPs were treated with 5 µg/mL AMD3100 for three days, fixed and stained. (A) Quantification of nestin and β-III tubulin staining showed no significant difference between AMD3100 treated and untreated cultures. (B) Levels of activated caspase 3, a marker for apoptosis, were similar between the two groups, and there were no differences between the NSC and immature neural populations as well.
(TIF)

Figure S5 Prior seizure experience leads to lower expression of the astrocyte marker glutamine synthetase. Glutamine synthetase (red, A-D) is expressed mainly by astrocytes. ESNPs (green, A-D) transplanted into mice with no prior seizure experience (PBS, A-B) show greater fluorescence intensity for glutamine synthetase than ESNPs grafted into mice with prior seizure experience (SE, C-D). (E) Normalizing fluorescence intensity of glutamine synthetase in ESNP grafts (yellow) to the PBS group, both the SE and the AMD3100 treated group (SE+AMD) had significantly lower fluorescence intensity. (F) As seizures are known to induce the activation and proliferation of astrocytes, the fluorescence intensity of glutamine synthetase in graft and non-graft areas were compared. Grafts of ESNPs showed greater average intensity of glutamine synthetase staining than the area surrounding the graft, and this ratio was significantly higher than both the SE and the SE+AMD groups.
(TIF)
