# Supplementary figures and images for: CXCL12-Mediated Guidance of Migrating Embryonic Stem Cell-Derived Neural Progenitors Transplanted into the Hippocampus
Source: PLoS One. 2010 Dec 31;5(12):e15856. doi: 10.1371/journal.pone.0015856 (PMC3013129; doi:10.1371/journal.pone.0015856)

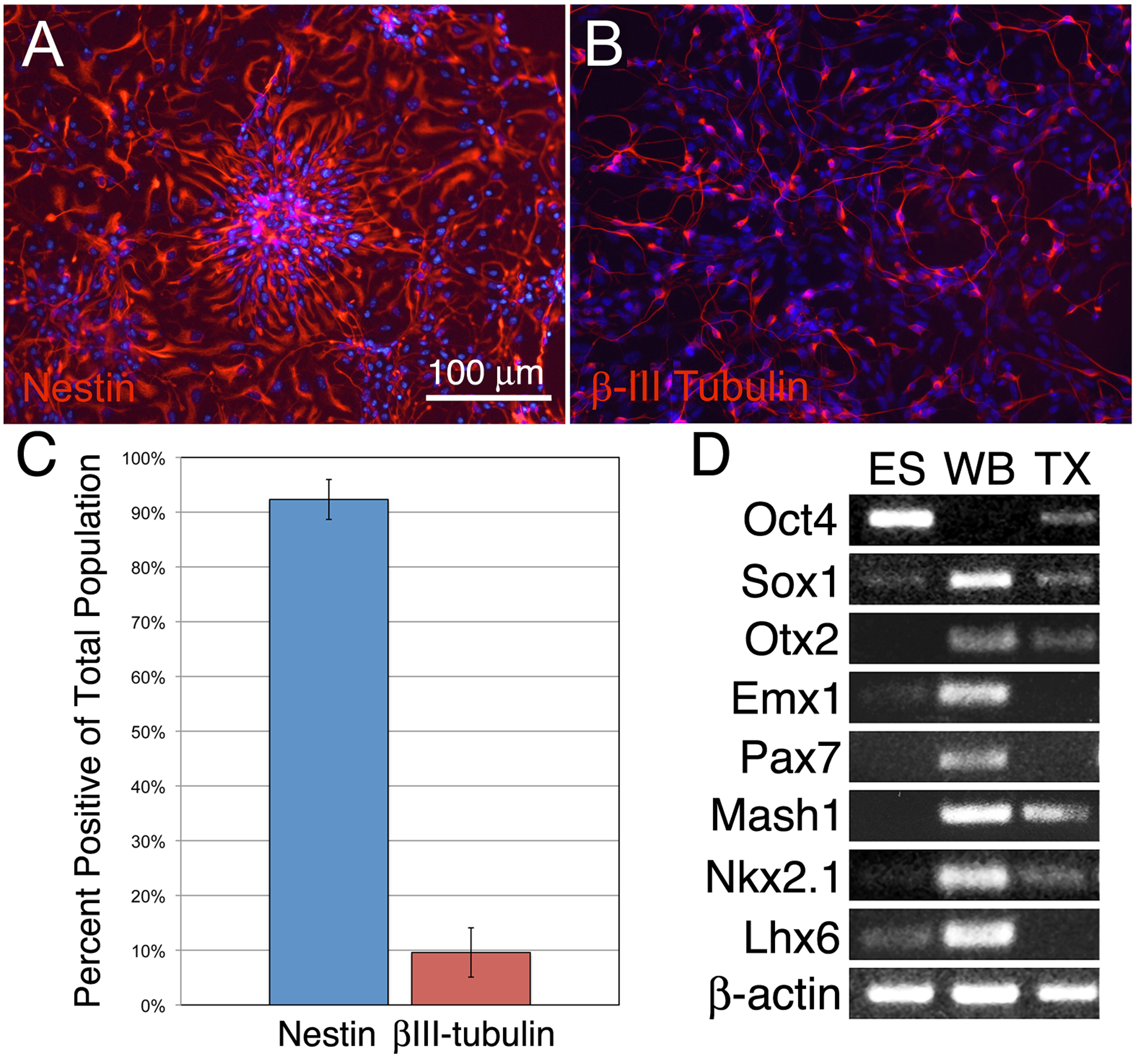

Supplement: Figure S1 — Characterization of ESNPs. Prior to transplantation, ESNPs expressed robust nestin (red, A) and β-III tubulin staining (red, B). (C) Quantification of immunofluorescent staining showed that over 92% of transplanted ESNPs expressed nestin, with fewer than 10% of the cells adopting neural fates, as shown by expression of the immature neuronal marker β-III tubulin. (D) RT-PCR analysis of YC5 embryonic stem cells (ES), E12.5 whole brain lysates (WB) and transplanted ESNPs (TX) showed that transplanted cells expressed markers for early NSCs (Sox1, Otx2), ventral NSCs (Mash1, Nkx2.1) and pluripotency (Oct4), albeit at a lower levels than ES cells. Primer sequences were as follows: Oct4 5′- CTCGAACCACATCCTTCTCT -3′ and 5′- GGCGTTCTCTTTGGAAAGGTGTTC -3′, Sox1: 5′- AATCCCCTCTCAGACGGTG -3′ and 5′- TTGATGCATTTTGGGGGTAT -3′, Otx2: 5′- AGGAGCTGAGTCGCCACCTC -3′ and 5′- GTAGCCCAGGGAGGGATGCA -3′, Emx1: 5′- AGCGACGTTCCCCAGGACGGGCTGC -3′ and 5′- CTGAGGTCACTTGGTC -3′, Pax7: 5′ – CCGTGTTTCTCATGGTTGTG -3′ and 5′- GAGCACTCGGCTAATCGAAC -3′, Mash1: 5′- CGACAGGACGCCCGCCTGAAAG -3′ and 5′- CTCGTCCTCTCCGGAACTGATG -3′, Nkx2.1 5′- AACAGCGGCCATGCAGCAGCAC -3′ and 5′- CCATGTTCTTGCTCACGTCC -3′, and Lhx6: 5′- CGACGACATCCACTACTCTCCGT -3′ and 5′- CAAGCTGAATTCGCCATTGCTCC -3′. (TIF) [file pone.0015856.s001.tif]

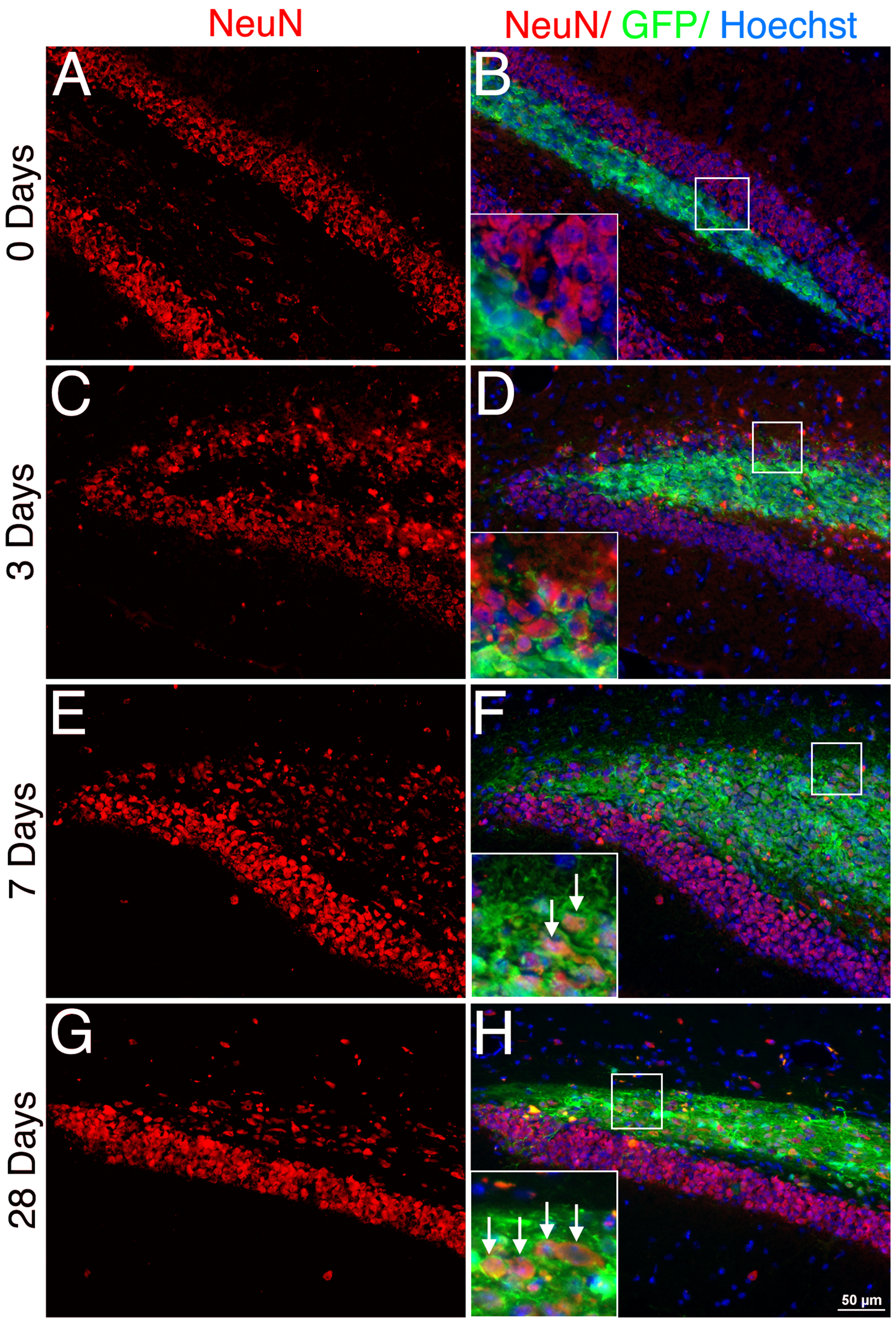

Supplement: Figure S2 — Endogenous DGNs degenerated within one week following ESNP transplantation to the upper blade. (A,B) Immediately following transplantation, NeuN+ neurons (red) in the upper blade were intact, and transplanted ESNPs (green) were clustered at the site of injection. (C,D) Within three days, significant degeneration of endogenous neurons in the upper blade was observed. (E,F) By one week, transplanted ESNPs began to express NeuN (arrows, inset) and degeneration of the upper blade continued. (G,H) By four weeks following transplantation, more engrafted cells were expressing NeuN (arrows, inset), and no more degeneration was observed. Nuclei are counterstained by Hoechst (blue). (TIF) [file pone.0015856.s002.tif]

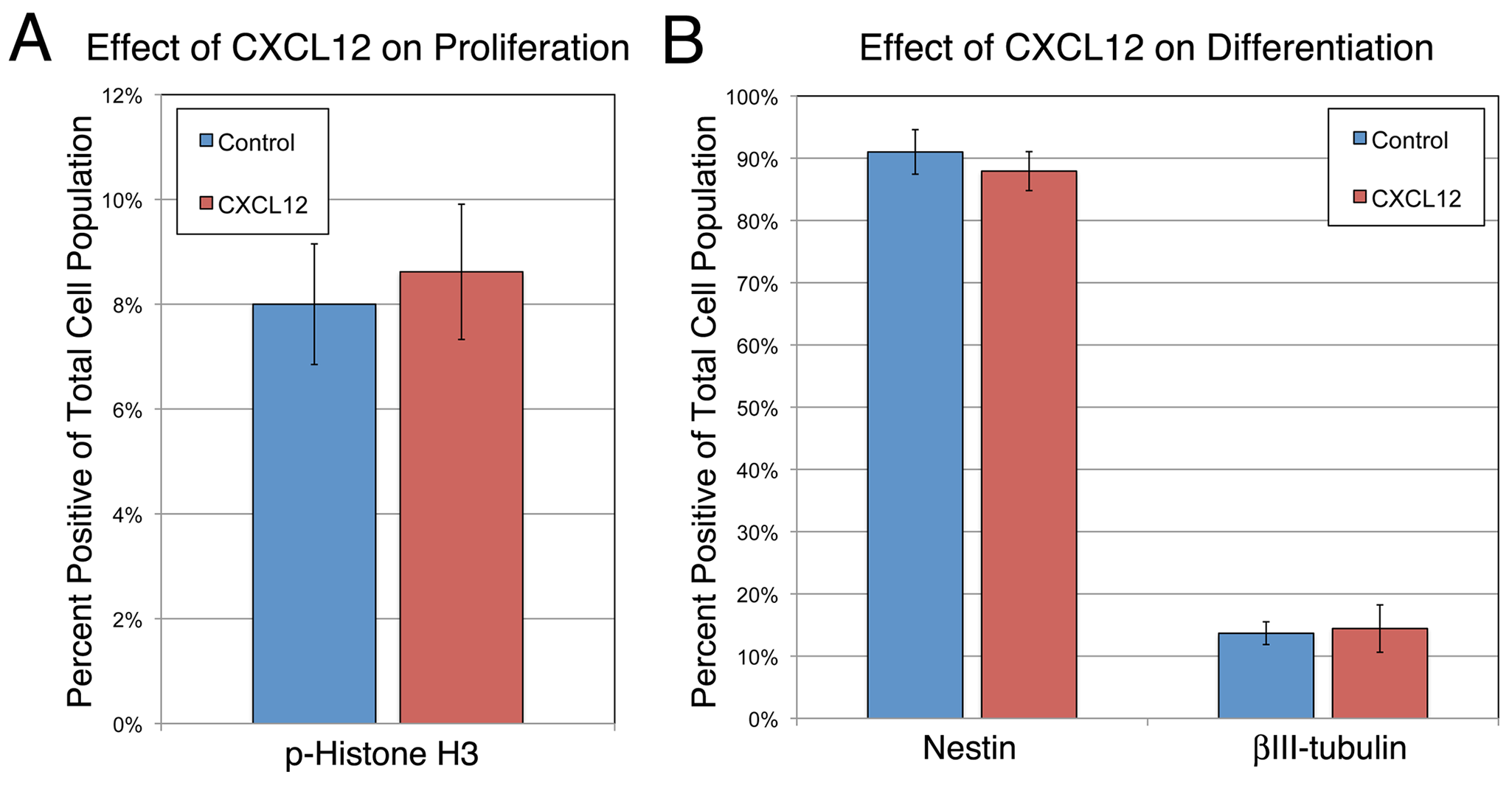

Supplement: Figure S3 — CXCL12 did not alter the proliferation or differentiation of YC5 ESNPs. YC5 ESNPs were treated with 200 ng/mL CXCL12 for three days, fixed and stained for markers of proliferation and differentiation. (A) Quantification of phospho-histone H3 revealed no significant difference between CXCL12 treated and untreated ESNPs. (B) Quantification showed similar levels of nestin and β–III tubulin staining after treatment with CXCL12 as compared to controls. (TIF) [file pone.0015856.s003.tif]

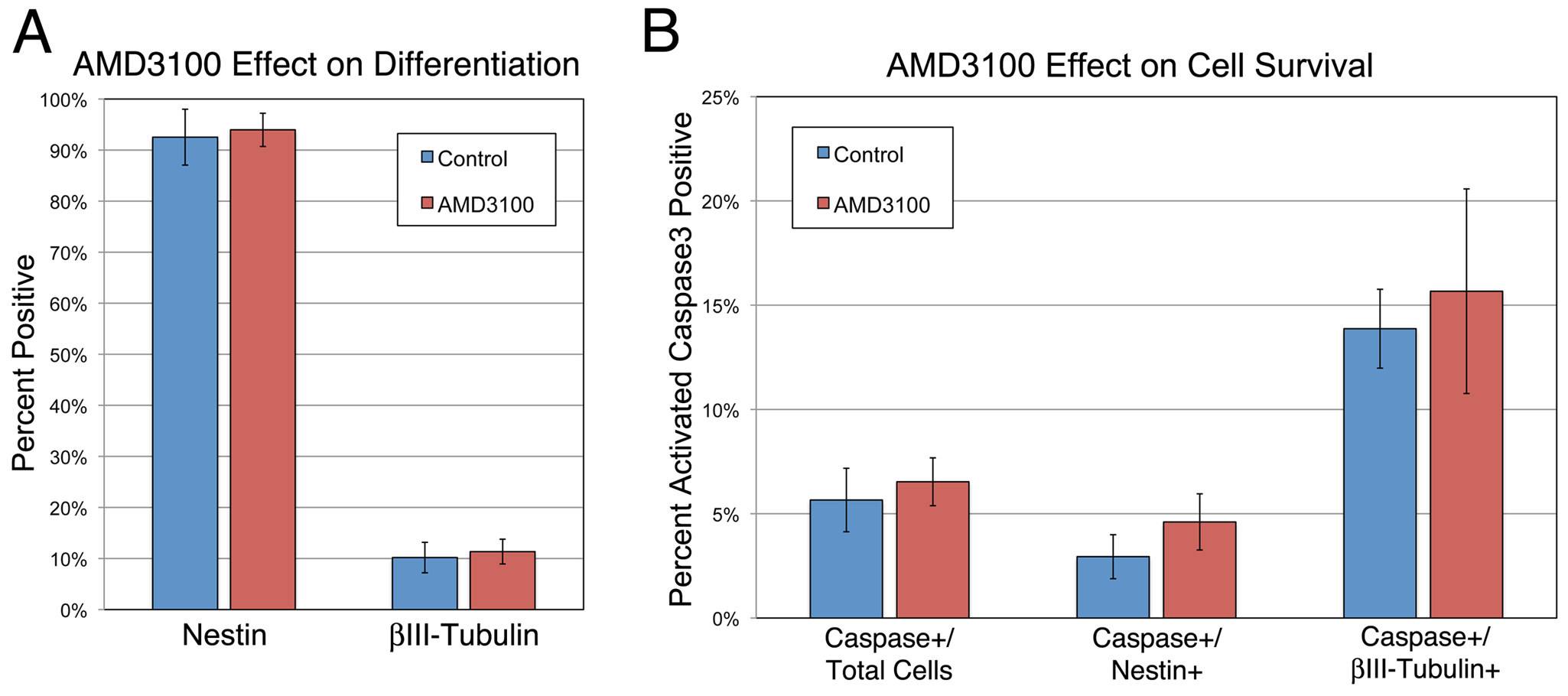

Supplement: Figure S4 — AMD3100 did not alter the differentiation or survival of ESNPs. YC5 ESNPs were treated with 5 µg/mL AMD3100 for three days, fixed and stained. (A) Quantification of nestin and β-III tubulin staining showed no significant difference between AMD3100 treated and untreated cultures. (B) Levels of activated caspase 3, a marker for apoptosis, were similar between the two groups, and there were no differences between the NSC and immature neural populations as well. (TIF) [file pone.0015856.s004.tif]

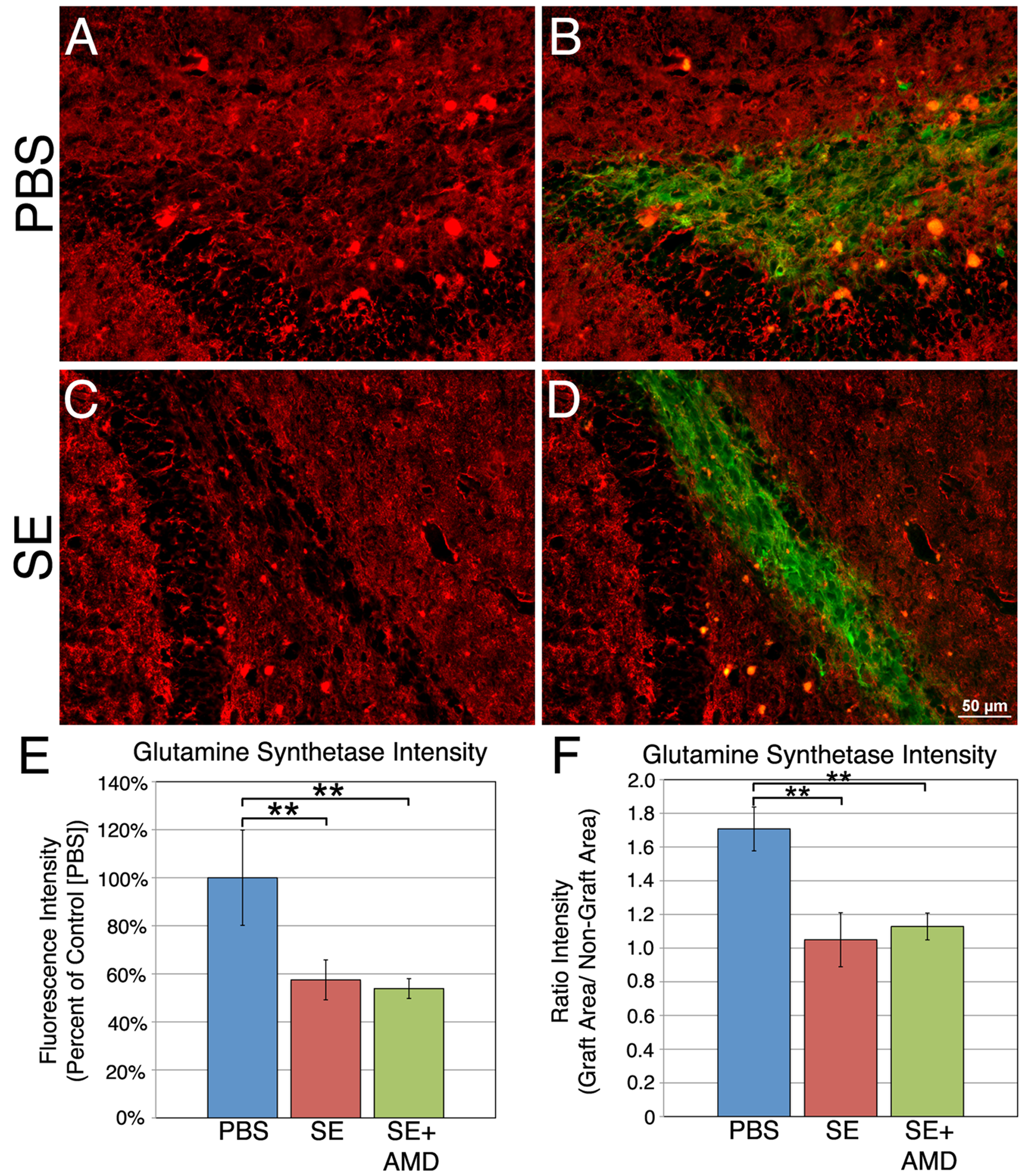

Supplement: Figure S5 — Prior seizure experience leads to lower expression of the astrocyte marker glutamine synthetase. Glutamine synthetase (red, A-D) is expressed mainly by astrocytes. ESNPs (green, A-D) transplanted into mice with no prior seizure experience (PBS, A-B) show greater fluorescence intensity for glutamine synthetase than ESNPs grafted into mice with prior seizure experience (SE, C-D). (E) Normalizing fluorescence intensity of glutamine synthetase in ESNP grafts (yellow) to the PBS group, both the SE and the AMD3100 treated group (SE+AMD) had significantly lower fluorescence intensity. (F) As seizures are known to induce the activation and proliferation of astrocytes, the fluorescence intensity of glutamine synthetase in graft and non-graft areas were compared. Grafts of ESNPs showed greater average intensity of glutamine synthetase staining than the area surrounding the graft, and this ratio was significantly higher than both the SE and the SE+AMD groups. (TIF) [file pone.0015856.s005.tif]
